# Association of Maternal Gestational Weight Gain With Left Ventricle Geometry and Function in Offspring at 4 Years of Age: A Prospective Birth Cohort Study

**DOI:** 10.3389/fped.2021.722385

**Published:** 2021-08-27

**Authors:** Jian Wang, Bowen Du, Yujian Wu, Zhuoyan Li, Qian Chen, Xi Zhang, Lin Zhang, Yujiao Ye, Yurong Wu, Sun Chen, Kun Sun

**Affiliations:** ^1^Department of Pediatric Cardiology, Xinhua Hospital, School of Medicine, Shanghai Jiao Tong University, Shanghai, China; ^2^Ministry of Education-Shanghai Key Laboratory of Childrens Environmental Health, Xinhua Hospital, School of Medicine, Shanghai Jiao Tong University, Shanghai, China; ^3^Clinical Research Unit, Xinhua Hospital, School of Medicine, Shanghai Jiao Tong University, Shanghai, China; ^4^Obstetrics Department, International Peace Maternity and Child Health Hospital of China, School of Medicine, Shanghai Jiao Tong University, Shanghai, China; ^5^Children Heart Center, Sichuan Provincial Maternity and Child Health Care Hospital, Sichuan, China

**Keywords:** gestational weight gain, left ventricle geometry, left ventricle hypertrophy, birth cohort, cardiovascular risk

## Abstract

**Background:** Maternal gestational weight gain (GWG) may be associated with cardiovascular diseases in the offspring from childhood to adulthood. We aimed to investigate the association between maternal GWG and the left ventricle (LV) geometry and function in the offspring, and explore the influence of the intrauterine environment on early childhood cardiac change.

**Methods:** Data of 981 mother-offspring pairs from the Shanghai Birth Cohort was used. Maternal pre-pregnancy weight and height, weight in the first trimester (≤ 12 weeks), and before delivery were measured. The echocardiography, blood pressure, and anthropometry assessment were evaluated in the offspring at 4 years of age.

**Results:** Interventricular septal thickness during diastole had a significantly positive correlation with total GWG [β = 0.009, (0.001, 0.017)]. In the second and third trimesters, LV mass index [β = 0.149, (0.015,0.282)], interventricular septal thickness in systole [β = 0.027, (0.011,0.043)], and in diastole [β = 0.014, (0.005,0.023)] were positively associated with GWG. The risks of eccentric [OR = 1.115, (1.232, 1.010)] and concentric hypertrophy [OR = 1.133, (1.259,1.018)] increased with the elevation of maternal GWG.

**Conclusions:** This study suggested that the excessive maternal GWG was associated with the thickening of the interventricular septum in the offspring, especially during the second and third trimesters. Excessive GWG in the second and third trimesters was a risk factor for LV eccentric and concentric hypertrophy in the offspring.

## Introduction

Left ventricle (LV) geometry and function are important factors that influence cardiac remodeling, and are also determinants of cardiovascular events in adulthood (1, 2). LV geometry may increase morbidity and mortality even in asymptomatic conditions, which could occur before the onset of overt hypertension and heart failure (3–6).

The LV geometry and functional changes are often evaluated in adults and adolescents. Morphologic changes in the LV could progress due to cumulative exposures from an early age, which could impair LV function ultimately in later life (3). However, studies on early LV geometry and functional changes in children are limited.

Apart from early influences during childhood, the LV geometry and function could also be affected by various maternal factors, such as obesity, gestational hypertension, and diabetes (7–9). Gestational weight gain (GWG) could reflect the health condition of both the fetus and mother during pregnancy (10, 11). It may play an important role in the development of cardiovascular diseases originating from the intrauterine environment (11–13). Increased GWG and maternal obesity may increase the risk of hypertension (14, 15), ventricular myocardial hypertrophy (16, 17), myocardial fibrosis (18), and congenital heart defects (19), which have been demonstrated in previous adult and animal studies. However, the impact of GWG on early LV geometry and function in young offspring remains unclear.

Based on the Shanghai Birth Cohort (SBC), we aimed to investigate the association of maternal GWG with offspring LV geometry and function at 4 years of age to explore the influence of the intrauterine environment on early childhood cardiac geometry and function.

## Materials and Methods

### Participants

The SBC is an ongoing prospective cohort study conducted in six collaborating hospitals in Shanghai, China. Volunteer couples were recruited during preconception care or in early pregnancy from 2013 to 2016, and the mother-fetus pairs were followed from preconception or early pregnancy to the end of the gestation. A detailed description of the cohort has been provided elsewhere (20). In our study, only women who had a singleton live birth, with recorded weight and height during pre-pregnancy, and recorded weight at pre-delivery were included. Miscarriage, stillbirth, multiple pregnancies, lost to follow-up, and women without available medical records were excluded. For offspring, children with congenital heart disease, lost to follow-up, uncooperative, and without available records were excluded. Finally, 981 mother-offspring pairs were included in the analysis. Ethical approval was granted by the Ethical Committee of Xinhua Hospital affiliated to Shanghai Jiao Tong University School of Medicine (Protocol no. XHEC-C-2013-001-2). All parents or guardians of participants signed the written informed consent before enrollment.

### Measurement of Maternal Factors

Information on demographic and sociodemographic characteristics (e.g., maternal age, race, education level), reproductive characteristics (e.g., parity, gestational week, delivery mode, birth weight and length), lifestyle factors (e.g., passive smoking or alcohol drinking status during pregnancy), and history of gestational hypertension or diabetes were collected through structured questionnaires and extraction of the inpatient history of the pregnant women from medical records. Pre-pregnancy weight and height, weight in the first trimester (≤ 12 weeks), and weight before delivery were measured at each clinical visit. Maternal pre-pregnancy body mass index (BMI), total GWG, GWG in the first (≤ 12 weeks) or second and third trimesters (>12 weeks) were calculated. Based on the World Health Organization (WHO) criteria, the pre-pregnancy BMI was categorized as underweight (< 18.5 kg/m^2^), normal weight (18.5–24.9 kg/m^2^), overweight (25.0–29.9 kg/m^2^), and obese (≥ 30.0 kg/m^2^) (21).

### Measurement of Offspring Factors

The height and weight of the offspring were measured according to the standard protocol. BMI was calculated in the 4-year-old children. Blood pressure (BP) and heart rate (HR) of the 4-year-old children were assessed by one trained staff while they were supine using the OMRAN HBP-1300 automatic BP device (Omron Healthcare, Guangzhou, China) on the left arm at heart level and with the appropriate cuff size for arm circumference. Three measurements were taken at 5-min intervals. The mean of the measurements was used in all analyses.

Transthoracic echocardiography examinations were performed for the children according to the American and European guidelines (22) by trained operators using the Philip EPIQ7C (Philips Healthcare, Andover, USA) ultrasound that uses the X5-1 (1-5MHz) or S8-3 (8-3MHz) matrix-array transducers (Philips Healthcare, Andover, USA). Measurements of the left ventricle (LV) dimensions were acquired from two-dimensional (2D)-guided M-mode echocardiograms, including the thickness of LV interventricular septum (IVSs and IVSd), posterior wall (LVPWs and LVPWd), and the internal diameter (LVIDs and LVIDd) of the LV during systole and diastole. LV ejection fraction (LVEF) and fractional shortening (FS) were calculated to evaluate the systolic function of LV.

Relative wall thickness (RWT) was calculated by the sum of the thickness of the LV posterior wall in diastole (LVPWd) and interventricular septal thickness in diastole (IVSd), then divided by the internal LV diameter in diastole (LVDd) (23). LV mass (LVM) was calculated using the Devereux formula (24) and the LVM index (LVMI) was calculated using the formula: LVMI = LVM/Height^2.7^ (25).

Pulse wave Doppler was used to measure the mitral early diastolic flow velocity (E), late diastolic flow velocity (A) to calculate the E/A ratio. The Doppler time intervals, including the ejection time (ET), isovolumic contraction time (ICT), and isovolumic relaxation time (IRT), were obtained at the mitral inflow and LV outflow tracts to calculate the Tei index, with the formula: Tei index = (ICT + IRT)/ET. These indices were used to assess the diastolic function of the LV.

For 2D speckle-tracking analysis, 2D images of 3–5 cardiac cycles were collected and analyzed with the commercial Qlab version 10.5 software (Philips Healthcare, Andover, USA)] at a frame rate of ≥ 60/s. Peak longitudinal strain was measured in the apical four, two, and three-chamber and global peak longitudinal strain (GLS) was calculated using the strain value of each segment in LV.

The LV geometry patterns were defined using the LVMI and RWT according to Ganau and colleagues' descriptions (5). There were no recommended LVMI and RWT cutoff points (26) for 4-year-old healthy children in China. We used the sex-specific 95th percentiles of LVMI and RWT derived from the cohort as cutoff points. LVMI = 33.24 g/m^2.7^, RWT = 0.27 in girls and LVMI = 33.76 g/m^2.7^, RWT = 0.27 in boys represented the sex-specific 95th percentiles in the cohort. Four groups were constructed for LV geometry patterns: (1) LVMI <33.24 g/m^2.7^ for girls and <33.76 g/m^2.7^ for boys, and RWT < 0.27 was classified as normal left ventricular geometry; (2) normal LVMI with increased RWT (>0.27) was classified as concentric remodeling; (3) increased LVMI (girls ≥33.24 g/m^2.7^, boys ≥ 33.76 g/m^2.7^), and normal RWT (<0.27) was defined as eccentric hypertrophy; and (4) increase in both variables was identified as concentric hypertrophy.

All the examinations were performed by a single experienced operator. Both the sonographers and the observers were blinded to the participants' details.

### Statistical Analyses

Linear regression models were used to investigate the associations between maternal GWG and offspring LV geometry and function changes. Five sets of models were constructed: the basic model was adjusted for none of the maternal factors or offspring factors. Model 1 was adjusted for maternal factors, including age at delivery, race, educational level, alcoholic drink intake history, exposure to passive smoke, pre-pregnancy BMI, gestational diabetes mellitus (GDM), and gestational hypertension or pre-eclampsia. Model 2 was adjusted additionally for gestational age, sex of offspring, delivery mode, and parity. Model 3 was adjusted additionally for BMI at 4 years of age. Model 4 was adjusted additionally for BP at 4 years of age. The GWG was divided into three groups according to the gestational trimesters as the total, first trimester, and second and third trimesters.

To further eliminate the effect of pre-pregnancy BMI on the LV geometry and function change in offspring, the study population was stratified into three groups (underweight, normal weight, and overweight or obese) according to the WHO criteria (21). Linear regression models were also constructed.

To test the risk of LV geometry pattern changes caused by an increase in GWG, including eccentric hypertrophy, concentric hypertrophy, and remodeling, multiple logistic regression analysis was performed in different groups, and odds ratio (OR) was calculated.

Statistical analysis was carried out using the SPSS 19.0 software program (IBM Corp., Armonk, NY, USA). All tests were two-sided with a significance level of 0.05.

## Results

### Basic Characteristics

The baseline characteristics of the study participants are presented in Supplementary Tables 1, 2. The mean total GWG was 14.4 ± 5.2 kg. On average, mothers gained a weight of 2.5 ± 3.2 kg in the first trimester and 11.9 ± 4.4 kg in the second and third trimesters. Most pregnant women enrolled had normal BMI (73.2%) before pregnancy. There were 14.1% who were underweight and 12.7% who were overweight or obese. In the offspring, the majority were male (52.8% male vs. 47.2% female). The average results of the LV structure and function data in the offspring were all within the reference range.

### Maternal GWG and Offspring LV Geometry and Function

In the basic model (Supplementary Table 3), almost all the indicators of LV internal cavity and wall thickness had a positive correlation with total GWG. However, there was no significant association between GWG with the LV systolic and diastolic function indices at 4 years of age. After adjusting for other maternal or offspring factors (Tables 1–3) which could influence the LV geometry and function in children, only the IVSd [β = 0.009, (0.001, 0.017)] had a significant positive correlation with the total GWG. There was no significant correlation between the LV global function indices and total GWG found in any models.

**Table 1:** Association between maternal total GWG and offspring LV geometry and function.

	**Total GWG**			
	**Model 1**	**Model 2**	**Model 3**	**Model 4**
**LV Structure**				
LVMI	0.14(−0.006,0.214)	0.106(−0.007,0.218)	0.097 (−0.018,0.213)	0.096(−0.023,0.215)
LVPWs	0.018(0.004,0.031)	**0.019(0.005,0.032)**	0.008(−0.006,0.021)	0.010(−0.003,0.024)
LVPWd	0.012(−0.001,0.025)	0.012(−0.001,0.025)	0.005(−0.008,0.018)	0.002(−0.011,0.016)
LVDs	**0.045(0.017,0.074)**	**0.040(0.011,0.069)**	0.003(−0.025,0.030)	0.001(−0.027,0.029)
LVDd	**0.058(0.022,0.095)**	**0.051(0.014,0.088)**	−0.006(−0.040,0.028)	−0.006(−0.040,0.028)
IVSs	**0.022(0.009,0.037)**	**0.022(0.008,0.036)**	0.011(−0.003,0.025)	0.013(−0.001,0.027)
IVSd	**0.010(0.003,0.018)**	**0.011(0.004,0.019)**	**0.009(0.001,0.017)**	**0.009(0.001,0.017)**
RWT	0.001(−0.001,0.001)	0.001(−0.001,0.001)	0.001(−0.001,0.001)	0.001(−0.001,0.001)
**LV Function**				
E/a	−0.001(−0.005,0.004)	−0.002(−0.006,0.003)	−0.002(−0.006,0.003)	−0.002(−0.007,0.02)
Tei Index	0.001(−0.001,0.001)	0.001(−0.001,0.001)	0.001(−0.001,0.002)	0.001(−0.001,0.002)
EF	0.058(−0.052,0.169)	0.073(−0.038,0.183)	0.079(−0.034,0.193)	0.082(−0.037,0.201)
AP2 strain	−0.014(−0.068,0.040)	−0.014(−0.070,0.041)	0.001(−0.055,0.005)	0.002(−0.054,0.058)
AP3 strain	−0.052(−0.115,0.011)	−0.049(−0.113,0.015)	−0.031(−0.094,0.033)	−0.030(−0.095,0.036)
AP4 strain	−0.052(−0.115,0.011)	−0.001(−0.053,0.052)	0.013(−0.039,0.066)	0.013(−0.041,0.067)
GLS	0.013(−0.041,0.067)	−0.021(−0.067,0.023)	−0.006(−0.051,0.038)	−0.005(−0.051,0.040)

*The bold values mean the mean difference is significant (P > 0.05)*.

*Model 1: adjusted for maternal factors, including age, race, educational level, drinking, passive smoking, pre-pregnancy BMI, GDM and gestational hypertension*.

*Model 2: adjusted additionally for gestational age, sex of offspring, delivery mode, parity*.

*Model 3: adjusted additionally for BMI at 4 years of age*.

*Model 4: adjusted additionally for BP at 4 years of age*.

*AP2 strain, peak longitudinal strain measured on apical two chambers; AP3 strain, peak longitudinal strain measured on apical three chambers; AP4 strain, peak longitudinal strain measured on apical four chambers, BMI, body mass index; EF, ejection fraction; GDM, gestational diabetes mellitus; GLS, global peak longitudinal strain; GWG, gestational weight gain; IVS, ventricle interventricular septal; IVSs, ventricle interventricular septal in systole; IVSd, ventricle interventricular septal in diastole; LVH, left ventricle hypertrophy; LVMI, LV mass index; LVPWd, LV posterior wall in diastole; LVPWs, LV posterior wall in systole: LVDd, LV diameter in diastole; LVDs, LV diameter in systole; RWT, relative wall thickness*.

**Table 2:** Association between maternal GWG and offspring LV geometry and function in the first trimester.

	**First trimester GWG**				
	**Model 1**	**Model 2**	**Model 3**	**Model 4**
**LV Structure**				
LVMI	−0.027(−0.200,0.146)	−0.012(−0.186,0.161)	−0.028(−0.203,0.146)	−0.039(−0.220,0.141)
LVPWs	0.0189(−0.002,0.040)	0.020(−0.002,0.041)	0.010(−0.011,0.031)	0.011(−0.010,0.033)
LVPWd	**0.014(0.001,0.027)**	**0.014(0.001,0.027)**	0.009(−0.004,0.022)	0.009(−0.005,0.023)
LVDs	0.018(−0.027,0.063)	0.024(−0.021,0.069)	−0.009(−0.050,0.033)	−0.009(−0.051,0.034)
LVDd	0.001(−0.056,0.059)	0.011(−0.046,0.069)	−0.039(−0.090,0.011)	−0.042(−0.03,0.010)
IVSs	−0.002(−0.024,0.020)	−0.001(−0.022,0.021)	−0.011(−0.032,0.010)	−0.012(−0.033,0.009)
IVSd	0.001(−0.011,0.012)	0.001(−0.011,0.012)	−0.002(−0.014,0.010)	**0.003(0.001,0.015)**
RWT	0.001(−0.001,0.001)	0.001(−0.001,0.001)	0.001(−0.001,0.001)	0.001(−0.001,0.001)
**LV Function**				
E/a	−0.006(−0.142,0.130)	−0.006(−0.144,0.132)	0.006(−0.132,0.144)	0.002(−0.137,0.141)
Tei Index	−0.001(−0.007,0.007)	0.001(−0.007,0.007)	−0.001(−0.007,0.007)	−0.001(−0.007,0.006)
EF	0.001(−0.001,0.001)	0.001(−0.001,0.002)	0.001(−0.001,0.002)	0.001(−0.001,0.002)
AP2 strain	−0.013(−0.096,0.069)	−0.005(−0.088,0.079)	0.009(−0.074,0.092)	0.009(−0.074,0.093)
AP3 strain	−0.022(−0.117,0.074)	−0.018(−0.115,0.079)	0.001(−0.096,0.096)	−0.012(−0.110,0.085)
AP4 strain	0.048(−0.030,0.126)	0.047(−0.032,0.126)	0.060(−0.018,0.138)	0.067(−0.013,0.147)
GLS	0.004(−0.063,0.071)	0.008(−0.060,0.075)	0.023(−0.044,0.089)	0.021(−0.047,0.089)

*The bold values mean the mean difference is significant (P > 0.05)*.

*Model 1: adjusted for maternal factors, including age, race, educational level, drinking, passive smoking, pre-pregnancy BMI, GDM and gestational hypertension*.

*Model 2: adjusted additionally for gestational age, sex of offspring, delivery mode, parity*.

*Model 3: adjusted additionally for BMI at 4 years of age*.

*Model 4: adjusted additionally for BP at 4 years of age*.

*AP2 strain, peak longitudinal strain measured on apical two chambers; AP3 strain, peak longitudinal strain measured on apical three chambers; AP4 strain, peak longitudinal strain measured on apical four chambers, BMI, body mass index; EF, ejection fraction; GDM, gestational diabetes mellitus; GLS, global peak longitudinal strain; GWG, gestational weight gain; IVS, ventricle interventricular septal; IVSs, ventricle interventricular septal in systole; IVSd, ventricle interventricular septal in diastole; LVH, left ventricle hypertrophy; LVMI, LV mass index; LVPWd, LV posterior wall in diastole; LVPWs, LV posterior wall in systole: LVDd, LV diameter in diastole; LVDs, LV diameter in systole; RWT, relative wall thickness*.

**Table 3:** Association between maternal GWG and offspring LV geometry and function in the second and third trimesters.

	**Second and third trimesters GWG**				
	**Model 1**	**Model 2**	**Model 3**	**Model 4**
**LV Structure**				
LVMI	**0.154(0.027,0.280)**	**0.155(0.026,0.284)**	**0.146(0.017,0.276)**	**0.149(0.015,0.282)**
LVPWs	0.009(−0.007,0.025)	0.009(−0.007,0.025)	0.003(−0.013,0.018)	0.006(−0.010,0.022)
LVPWd	0.005(−0.005,0.015)	0.006(−0.004,0.015)	0.002(−0.007,0.012)	0.003(−0.007,0.013)
LVDs	**0.043(0.010,0.076)**	**0.037(0.003,0.070)**	0.015(−0.016,0.046)	0.010(−0.022,0.041)
LVDd	**0.063(0.021,0.105)**	**0.054(0.011,0.096)**	0.021(−0.017,0.059)	0.018(−0.020,0.056)
IVSs	**0.031(0.015,0.046)**	**0.031(0.015,0.047)**	**0.024(0.008,0.039)**	**0.027(0.011,0.043)**
IVSd	**0.013(0.004,0.021)**	**0.015(0.006,0.023)**	**0.013(0.005,0.022)**	**0.014(0.005,0.023)**
RWT	0.001(−0.001,0.001)	0.001(−0.001,0.001)	0.001(−0.001,0.001)	0.001(−0.001,0.001)
**LV Function**				
E/a	0.001(−0.004,0.006)	0.001(−0.004,0.006)	0.001(−0.004,0.007)	0.001(−0.005,0.006)
Tei Index	0.001(−0.001,0.002)	0.001(−0.001,0.002)	0.001(−0.001,0.002)	0.001(−0.001,0.002)
EF	0.010(−0.091,0.112)	0.009(−0.096,0.114)	0.019(−0.086,0.124)	0.030(−0.077,0.136)
AP2 strain	−0.011(−0.072,0.051)	−0.016(−0.080,0.048)	−0.005(−0.068,0.058)	−0.003(−0.067,0.051)
AP3 strain	−0.055(−0.126,0.016)	−0.054(−0.128,0.020)	−0.040(−0.113,0.033)	−0.031(−0.107,0.044)
AP4 strain	−0.017(−0.075,0.041)	−0.028(−0.088,0.032)	−0.018(−0.078,0.042)	−0.023(−0.084,0.039)
GLS	−0.028(−0.078,0.021)	−0.033(−0.085,0.018)	−0.022(−0.072,0.029)	−0.020(−0.072,0.032)

*The bold values mean the mean difference is significant (P > 0.05)*.

*Model 1: adjusted for maternal factors, including age, race, educational level, drinking, passive smoking, pre-pregnancy BMI, GDM, and gestational hypertension*.

*Model 2: adjusted additionally for gestational age, sex of offspring, delivery mode, parity*.

*Model 3: adjusted additionally for BMI at 4 years of age*.

*Model 4: adjusted additionally for BP at 4 years of age*.

*AP2 strain, peak longitudinal strain measured on apical two chambers; AP3 strain, peak longitudinal strain measured on apical three chambers; AP4 strain, peak longitudinal strain measured on apical four chambers; BMI, body mass index; EF, ejection fraction; GDM, gestational diabetes mellitus; GLS, global peak longitudinal strain; GWG, gestational weight gain; IVS, ventricle interventricular septal; IVSs, ventricle interventricular septal in systole; IVSd, ventricle interventricular septal in diastole; LVH, left ventricle hypertrophy; LVMI, LV mass index; LVPWd, LV posterior wall in diastole; LVPWs, LV posterior wall in systole; LVDd, LV diameter in diastole; LVDs, LV diameter in systole; RWT, relative wall thickness*.

The gestational time was divided into the total, first trimester (≤ 12 weeks), and second and third trimesters (>12 weeks). In the basic model, LVMI [β = 0.147, (0.027, 0.266)], LVDd [β = 0.051, (0.011, 0.091)], IVSd [β = 0.033, (0.017, 0.047)], and IVSs [β = 0.014, (0.006, 0.022)] had a positive association with the second and third trimester GWG. GWG in the first trimester had no significant correlation with any structural and functional indices. After adjusting for maternal and offspring factors, LVMI [β = 0.149, (0.015, 0.282)], IVSs [β = 0.027, (0.011, 0.043)], and IVSd [β = 0.014, (0.005, 0.023)] continued to be positively associated with GWG in all models during the second and third trimesters. The RWT and the LV function indices had no significant correlation with GWG in any trimester.

### Offspring LV Geometry and Function in Different Pre-pregnancy BMI Groups

GWG was strongly related to pre-pregnancy BMI (27). Taking the pre-pregnancy BMI into consideration, we divided the maternal-offspring pairs into three groups (underweight, normal weight, and overweight or obese) to eliminate the influence of pre-pregnancy BMI (Table 4). For underweight women, LVPWs was directly associated with the total [β = 0.046, (0.001, 0.091)] and the first trimester GWG [β = 0.068, (0.001, 0.135)]; LVPWd was positively associated with the total [β = 0.035, (0.007, 0.062)] and the second and third trimester GWG [β = 0.035, (0.002, 0.067)]; IVSs was associated with the total GWG [β = 0.049, (0.002, 0.095)]. For normal-weight women, IVSd were positively correlated with the total [β = 0.012, (0.003, 0.022)], and the second and third GWG [β = 0.015, (0.004, 0.026)]; IVSs had a positive correlation with the second and third trimester GWG [β = 0.020, (0.001, 0.039)]. But in overweight and obese women, only IVSs were correlated with the second and third trimester GWG. There was no significant association of LV function indices with GWG in any of the groups.

**Table 4:** Association between maternal GWG and LV geometry and function in offspring divided by pre-pregnant BMI.

	**Total GWG**			**First trimester GWG**			**Second and third trimesters GWG**		
	**Underweight**	**Normal weight**	**Overweight and obese**	**Underweight**	**Normal weight**	**Overweight and obese**	**Underweight**	**Normal weight**	**Overweight and obese**
**LV Structure**									
LVMI	0.173 (−0.243,0.588)	0.137 (−0.004,0.279)	−0.037 (−0.305,0.230)	−0.150 (−0.770,0.469)	0.073 (−0.152,0.297)	−0.264 (−0.632,0.105)	0.321 (−0.155,0.797)	0.144 (−0.016,0.305)	0.168 (−0.180,0.517)
LVPWs	**0.046** **(0.001,0.091)**	0.008 (−0.009,0.025)	−0.003 (−0.029,0.023)	**0.068** **(0.001,0.135)**	0.010 (−0.017,0.038)	−0.001 (−0.037,0.036)	0.020 (−0.033,0.074)	0.005 (−0.015,0.024)	−0.004 (−0.038,0.030)
LVPWd	**0.035** **(0.007,0.062)**	0.007 (_0.003,0.019)	−0.007 (−0.025,0.011)	0.018 (−0.024,0.061)	0.013 (−0.004,0.030)	−0.003 (−0.028,0.022)	**0.035** **(0.002,0.067)**	0.004 (−0.009,0.016)	−0.009 (−0.033,0.014)
LVDs	−0.025 (−0.127,0.078)	0.015 (−0.017,0.048)	−0.032 (−0.099,0.036)	0.045 (−0.107,0.197)	0.006 (−0.045,0.058)	−0.080 (−0.173,0.012)	−0.059 (−0.177,0.059)	0.016 (−0.021,0.053)	0.017 (−0.072,0.105)
LVDd	−0.009 (−0.146,0.128)	−0.008 (−0.047,0.032)	−0.013 (−0.096,0.070)	0.042 (−0.161,0.245)	−0.034 (−0.096,0.028)	−0.096 (−0.210,0.018)	−0.037 (−0.195,0.121)	0.010 (−0.035,0.054)	0.062 (−0.046,0.171)
IVSs	**0.049** **(0.002,0.095)**	0.009 (−0.007,0.026)	0.012 (−0.019,0.043)	0.025 (−0.045,0.095)	−0.011 (−0.038,0.015)	−0.027 (−0.071,0.016)	0.048 (−0.006,0.102)	**0.020** **(0.001,0.039)**	**0.045** **(0.005,0.085)**
IVSd	0.011 (−0.019,0.040)	**0.012** **(0.003,0.022)**	0.006 (−0.011,0.022)	−0.016 (−0.060,0.027)	0.002 (−0.013,0.017)	−0.001 (−0.024,0.022)	0.024 (−0.010,0.057)	**0.015** **(0.004,0.026)**	0.010 (−0.011,0.032)
RWT	0.001 (−0.001,0.003)	**0.001** **(0.000,0.002)**	−0.001 (−0.001,0.001)	−0.001 (−0.003,0.002)	0.001 (−0.001,0.001)	0.001 (−0.001,0.002)	0.002 (−0.001,0.004)	0.001 (−0.001,0.001)	−0.001 (−0.002,0.001)
**LV Function**									
E/a	0.002 (−0.016,0.201)	−0.001 (−0.007,0.005)	−0.003 (−0.014,0.007)	−0.013 (−0.037,0.010)	0.008 (−0.008,0.009)	−0.001 (−0.014,0.013)	0.012 (−0.008,0.031)	−0.001 (−0.007,0.006)	−0.005 (−0.019,0.008)
EF	0.177 (−0.174,0.528)	0.041 (−0.070,0.151)	−0.137 (−0.370,0.096)	0.209 (−0.097,0.515)	−0.039 (−0.225,0.147)	−0.171 (−0.479,0.136)	−0.090 (−0.437,0.256)	0.073 (−0.055,0.200)	−0.061 (−0.358,0.236)
Tei Index	0.001 (−0.003,0.004)	0.001 (−0.001,0.002)	−0.001 (−0.002,0.002)	0.001 (−0.004,0.006)	0.001 (−0.002,0.002)	0.001 (−0.003,0.004)	0.001 (−0.004,0.004)	0.001 (−0.001,0.002)	−0.001 (−0.003,0.003)
AP2 strain	−0.015 (−0.234,0.205)	−0.007 (−0.074,0.060)	0.040 (−0.114,0.194)	−0.108 (−0.299,0.082)	−0.019 (−0.131,0.094)	0.136 (−0.063,0.335)	0.121 (−0.091,0.334)	0.001 (−0.077,0.078)	−0.060 (−0.253,0.133)
AP3 strain	0.150 (−0.104,0.404)	−0.072 (−0.151,0.007)	0.086 (−0.087,0.259)	0.019 (−0.207,0.244)	−0.084 (−0.217,0.050)	0.118 (−0.110,0.345)	0.121 (−0.129,0.371)	−0.057 (−0.149,0.035)	0.029 (−0.190,0.249)
AP4 strain	0.004 (−0.219,0.223)	0.020 (−0.043,0.084)	0.025 (−0.132,0.183)	0.063 (−0.133,0.258)	0.085 (−0.021,0.191)	0.108 (−0.098,0.314)	−0.074 (−0.292,0.144)	−0.014 (−0.087,0.060)	−0.058 (−0.255,0.140)
GLS	0.042 (−0.128,0.212)	−0.020 (−0.074,0.035)	0.049 (−0.082,0.179)	−0.012 (−0.162,0.138)	−0.005 (−0.096,0.087)	0.119 (−0.049,0.288)	0.055 (−0.111,0.222)	−0.024 (−0.087,0.039)	−0.032 (−0.196,0.133)

*The bold values mean the mean difference is significant (P > 0.05)*.

*Linear regression was adjusted for maternal and offspring factors, including maternal age, race, educational level, drinking, passive smoking, pre-pregnancy BMI, GDM and gestational hypertension, gestational age, sex of offspring, delivery mode, parity, BMI, and BP of offspring at 4 years of age*.

*AP2 strain, peak longitudinal strain measured on apical two chambers; AP3 strain, peak longitudinal strain measured on apical three chambers; AP4 strain, peak longitudinal strain measured on apical four chambers; BMI, body mass index; EF, ejection fraction; GDM, gestational diabetes mellitus; GLS, global peak longitudinal strain; GWG, gestational weight gain; IVS, ventricle interventricular septal; IVSs, ventricle interventricular septal in systole; IVSd, ventricle interventricular septal in diastole; LVH, left ventricle hypertrophy; LVMI, LV mass index; LVPWd, LV posterior wall in diastole; LVPWs, LV posterior wall in systole; LVDd, LV diameter in diastole; LVDs, LV diameter in systole; RWT, relative wall thickness*.

### Risk of Left Ventricle Hypertrophy in Offspring at 4 Years of Age

**Figure 1:**
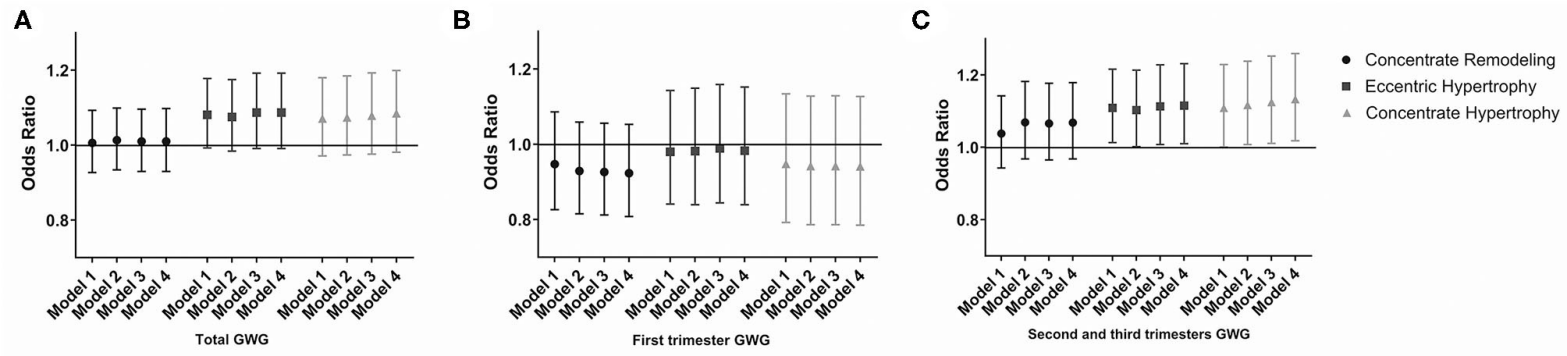
Odds risk (OR) of LVH in offspring with maternal GWG during different gestational trimesters. **(A)** OR of LVH in offspring with maternal GWG in total trimester. **(B)** OR of LVH in offspring with maternal GWG in first trimester. **(C)** OR of LVH in offspring with maternal GWG in second and third trimesters. Data are presented as OR (95%CI). Model 1: adjusted for maternal factors, including age, race, educational level, drinking, passive smoking, pre-pregnancy BMI, GDM and gestational hypertension. Model 2: adjusted additionally for gestational age, sex of offspring, delivery mode, parity. Model 3: adjusted additionally for BMI at 4 years of age. Model 4: adjusted additionally for BP at 4 years of age. BMI, body mass index; GWG, gestational weight gain; LVH, left ventricle hypertrophy.

The risk of four types of LV geometry change patterns in offspring with maternal GWG in different trimesters is presented in Figure 1. In the second and third trimesters, the risk of eccentric [OR = 1.115, (1.232, 1.010)] and concentric hypertrophy [OR = 1.133, (1.259, 1.018)] increased with an elevation of maternal GWG after adjusting for the maternal and offspring factors. Eccentric, concentric hypertrophy, and remodeling were three types of LVH. It indicated that excessive GWG in the second and third trimesters was an independent risk factor for LVH in the offspring.

## Discussion

In this prospective cohort study, we found that greater GWG was associated with LV morphologic changes in the offspring as early as 4 years of age, especially the thickening of IVS. Greater GWG during the second and third trimesters was an independent risk factor for LV eccentric and concentric hypertrophy in the offspring.

Cardiac structural changes usually occur before global functional alteration, which is often evaluated during adulthood and adolescence. However, morphologic changes in the LV could progress due to accumulated exposures during childhood, which could impair the LV function ultimately in later life (3). In our study, regional cardiac structural changes occurred as early as 4 years of age during follow-up, whereas there were no changes in the global function. This result provided evidence for early screening of the cardiovascular structure changes in young children, which may be useful for preventing the generation and progression of cardiovascular disease in adulthood.

LV wall thickening is the manifestation of myocardial hypertrophy (28). The thickness of the IVS is one of the reliable markers for evaluating the adverse outcomes in cardiovascular diseases, including coronary artery disease (29), atrial fibrillation (30), and valve replacement (31). In patients with hypertension, the thickening of IVS has been observed before LVMI and functional changes (32); conversely, isolated hypertrophy of the IVS with normal LVM and function has been demonstrated to increase the risk of the development of hypertension in the future (33–35). The thickness of IVS is a multifactorial index influenced by the growth and BP of children. In our study, the thickness of the IVS was found to be significantly associated with greater GWG, independent of offspring's BMI, BP, and other factors.

We focused on the LV geometry and functional patterns in a birth cohort comprising randomly selected children. These LV structural changes, including thickening of the IVS, may be physiologic during early childhood since the pathological changes usually occur in adolescents (26, 36) and adults (9), or in high-risk groups of children, such as those with obesity (37), obstructive sleep apnea (38), or hypertension (39). The transition to pathologic remodeling could be heralded by progressive ventricular dilatation, distortion of the shape of the cavity, and disruption of the normal cardiac geometry and function (40). The LV geometry patterns described the four different LV remodeling features. Eccentric hypertrophy, concentric hypertrophy, and cardiac remodeling were classified as different types and states of LVH. LVH indicated a pathological hyperdynamic state, which may alter LV structure and function that may predispose to the development of heart failure or other adverse cardiovascular prognosis (41). The pressure overload pattern of concentric hypertrophy was associated with high systolic blood pressure and high peripheral resistance. Eccentric LV hypertrophy was associated with normal peripheral resistance, but high cardiac indexes consistent with excess circulating blood volume. Concentric remodeling was characterized by high peripheral resistance, low cardiac index, and increased arterial stiffness (5, 40, 42). In Cuspidi's meta-analysis (43), the risk of LVH was 4.2-fold greater in obese than in non-obese participants and eccentric hypertrophy was the most common type of LVH in obesity. Consistently, we found that the maternal GWG during the second and third trimesters was an independent risk factor for hyperdynamic status in children at 4 years of age. It indicated that the maternal intrauterine environment may have a long-term influence on their offspring's hemodynamic status. However, this hypothesis needs further validation in future studies with larger sample sizes.

Greater GWG was associated with many adverse maternal and offspring outcomes from birth to adulthood. Greater maternal pre-pregnancy weight and GWG were associated with higher systolic blood pressure (36), adverse accumulation of lipid and inflammatory profiles (9, 44), increased risk of congenital heart disease (19, 45), myocardial hypertrophy (16), hypertension (15), and premature death in later life in the offspring (46, 47). The GWG in the second and third trimesters accounted for a majority of the total GWG and played an important role in offspring growth, adiposity, and metabolic state (48). It was a key period for organ development, including the heart, muscles, bones, and liver (48–50). In our study, greater GWG during the second and third trimesters was significantly associated with thickening of the LV wall and was a risk factor for LVH in the offspring, independent of the influence from offspring growth or BP. Therefore, judicious monitoring of GWG during the second and third trimesters should be recommended, which may help promote the health of both the mother and her child.

There are several possible explanations for the mechanisms responsible for a greater GWG increasing the risk of LVH. Greater maternal GWG is an abnormal metabolic state, which modifies the intrauterine environment, thereby influencing placental function and fetal programming of the cardiovascular system, which, in turn, affects fetal heart development. A mother with excessive weight gain or obesity has a suboptimal uterine nutritional climate, which may disrupt the inflammatory and hormone metabolic homeostasis of the mother, including insulin resistance, increased level of leptin, lipid, and pro-inflammatory cytokines. These metabolic disorders may change the placental hemodynamics causing placental vascular insufficiency, increased lipogenesis, infarction, hypoxia, and inflammatory activation (12). These may cause an abnormal accumulation of glycogen and glucose uptake in the myocytes, thus, increasing the load on the fetal heart, ultimately resulting in myocyte hypertrophy (28, 51). Furthermore, the abnormal intrauterine environment may influence the epigenetic modifications of genes associated with cardiovascular function and development (13). The effect of epigenetics is inborn and lifelong. It may have a cumulative effect on the vascular and myocyte function in the offspring from the fetal stage to adulthood. These may increase the risk of the early development of hypertension, ventricular hypertrophy, atherosclerosis, and premature cardiac failure, consequently influencing the prognosis of cardiovascular health in later life (12).

Early screening of LV geometry and function by echocardiography at 4 years of age without the administration of any sedative agents was feasible. Thus, this age may be a suitable time for early screening. Furthermore, early screening of LV structural changes should be recommended in the offspring of mothers who had excessive weight gain during pregnancy, and emphasize the need for additional monitoring and weight management during the second and third trimesters.

### Strengths and Limitations

To our knowledge, this study was the largest prospective birth cohort study that assessed LV geometry and function using detailed echocardiography during early childhood in China. Furthermore, previous research on the association between maternal and offspring factors associated with LV geometry usually focused on maternal obesity (46) and were performed in adolescents, adults, or high-risk groups of offspring with obesity (43), abnormal blood pressure (52), or other metabolic syndromes (26). This study provided the first evidence of maternal GWG as an independent risk factor for LV hypertrophy at an early age in the general population.

Our study has several limitations. First, cardiac magnetic resonance imaging is the gold standard to evaluate LV geometry. However, this procedure requires the administration of sedatives in young children. Therefore, in our study, we opted for echocardiography as a more suitable modality for children aged 4 years. Second, data on maternal weight during the second trimester were not available in the medical records, which may need a more detailed investigation in future studies. Third, there was an unequal representation of the populations enrolled in our study regarding the different degrees of GWG and pre-pregnancy BMI, which may have influenced the results of the subgroup analysis.

### Conclusion

Excessive GWG, especially during the second and third trimesters, was associated with increased thickening of the IVS. Thus, excessive GWG during the second and third trimesters is a risk factor for LV eccentric and concentric hypertrophy at 4 years of age in the offspring. Our results provide evidence supporting the early screening of LV geometry and function during early childhood, and emphasize the need for additional monitoring and weight management of pregnant mothers during the second and third trimesters.

## Data Availability Statement

The raw data supporting the conclusions of this article will be made available by the authors, without undue reservation.

## Ethics Statement

The studies involving human participants were reviewed and approved by the research ethics boards of Shanghai Xinhua Hospital (the coordination center, approved on August 23, 2013, ref no. M2013-010). Written informed consent to participate in this study was provided by the participants' legal guardian/next of kin.

## Author Contributions

JW, BD, and YW drafted and revised the manuscript. JW, BD, YW, ZL, YW, SC, and KS contributed to the conception and design of the work. JW, BD, YW, ZL, and YY contributed to the acquisition of data. JW, BD, YW, QC, XZ, and ZL contributed to the analysis or interpretation of the data. YW, SC, and KS critically revised the manuscript. All authors gave their final approval and agreed to be accountable for all aspects of this work ensuring its integrity and accuracy.

## Conflict of Interest

The authors declare that the research was conducted in the absence of any commercial or financial relationships that could be construed as a potential conflict of interest.

## Publisher's Note

All claims expressed in this article are solely those of the authors and do not necessarily represent those of their affiliated organizations, or those of the publisher, the editors and the reviewers. Any product that may be evaluated in this article, or claim that may be made by its manufacturer, is not guaranteed or endorsed by the publisher.
