# Supplementary material for: Association of Maternal Gestational Weight Gain With Left Ventricle Geometry and Function in Offspring at 4 Years of Age: A Prospective Birth Cohort Study
Source: Front Pediatr. 2021 Aug 27;9:722385. doi: 10.3389/fped.2021.722385 (PMC8429845; doi:10.3389/fped.2021.722385)
Supplement: Supplementary file 1 [file Table_1.docx]

**Supplementary Table 1. Baseline characteristic of the mother-offspring pairs.**

|  | N (%) | Mean (SD) |
| --- | --- | --- |
| Maternal characteristics | 981(100) |  |
| Age at pregnancy(years) |  | 30.8(3.4) |
| Race |  |  |
| Han | 961(98.0) |  |
| Others | 20(2.0) |  |
| Education level |  |  |
| Junior college or below | 277(28.2) |  |
| Undergraduate | 554(56.5) |  |
| Postgraduate | 150(15.3) |  |
| Pre-pregnancy BMI (kg/m^2^) |  |  |
| Underweight (<18.5) | 138(14.1) |  |
| Normal weight (18.6-24.9) | 718(73.2) |  |
| Overweight and Obese (>25.0) | 125(12.7) |  |
| Passive smoking during pregnancy |  |  |
| Yes | 271(27.6) |  |
| No | 710(72.4) |  |
| Drinking at pregnancy |  |  |
| Yes | 121(12.3) |  |
| No | 860(87.7) |  |
| Gestational weight gain(kg) |  |  |
| Total |  | 14.4(5.2) |
| First trimester |  | 2.5(3.2) |
| Second and third trimesters |  | 11.9(4.4) |
| Gestational Hypertension and pre-eclampsia | 77(7.8) |  |
| Gestational Diabetes | 127(12.9) |  |
|  |  |  |
| Offspring characteristics | 981(100) |  |
| Gender |  |  |
| male | 518(52.8) |  |
| female | 463(47.2) |  |
| Gestation week (weeks) |  |  |
| <37 | 56(5.7) |  |
| 37-42 | 885(90.3) |  |
| >42 | 39(4.0) |  |
| Delivery mode |  |  |
| Eutocia | 509(51.9) |  |
| Cesarean delivery | 276(28.1) |  |
| Parity |  |  |
| Nulliparas | 954(97.2) |  |
| Multiparas | 27(2.8) |  |
| Weight at birth (kg) |  | 3.3(0.5) |
| Length at birth (cm) |  | 49.8(1.4) |
| BMI at birth (kg/m^2^) |  | 13.5(1.5) |
| Weight at 4 years (kg) |  | 17.2(2.6) |
| Length at 4 years (cm) |  | 107.2(4.6) |
| BMI at 4 years (kg/m^2^) |  | 20.0(4.6) |
| SBP at 4 years (mmHg) |  | 98.1(7.8) |
| DBP at 4 years (mmHg) |  | 57.5(6.3) |
| HR at 4 years (bpm) |  | 91.9(10.4) |

Continuous variables are expressed as mean (±SD), and categorical variables are expressed as number [percentage (%)]

BMI: Body mass index; SBP: Systolic blood pressure; DBP: Diastolic blood pressure; HR: Heart rate; CI: Confidence interval; SD: Standard deviation
