# Supplementary material for: Association of Maternal Gestational Weight Gain With Left Ventricle Geometry and Function in Offspring at 4 Years of Age: A Prospective Birth Cohort Study
Source: Front Pediatr. 2021 Aug 27;9:722385. doi: 10.3389/fped.2021.722385 (PMC8429845; doi:10.3389/fped.2021.722385)
Supplement: Supplementary file 2 [file Table_2.docx]

**Supplementary Table 2. Baseline characteristic of LV structure and function in offspring.**

| Offspring characteristics |  | Mean (SD) |
| --- | --- | --- |
| LV structure |  |  |
| LVMI(g/m^2.7^) |  | 26.0(4.6) |
| LVPWs(mm) |  | 7.8(0.9) |
| LVPWd(mm) |  | 4.1(0.6) |
| LVDs(mm) |  | 22.6(1.9) |
| LVDd(mm) |  | 35.5(2.5) |
| IVSs(mm) |  | 6.5(0.9) |
| IVSs(mm) |  | 3.6(0.5) |
| RWT |  | 0.2(0.03) |
| LV function |  |  |
| E/a |  | 1.8(0.3) |
| Tei index |  | 0.4(0.1) |
| EF(%) |  | 59.7(4.7) |
| AP2 Strain(%) |  | 23.7(2.8) |
| AP3 Strain(%) |  | 23.6(3.3) |
| AP4 Strain(%) |  | 23.6(2.7) |
| GLS(%) |  | 23.6(2.3) |

Continuous variables are expressed as mean (±SD), and categorical variables are expressed as number [percentage (%)]

BMI: Body mass index; SBP: Systolic blood pressure; DBP: Diastolic blood pressure; HR: Heart rate; CI: Confidence interval; SD: Standard deviation;

AP2 Strain: peak longitudinal strain measured on apical two chambers; AP3 Strain: peak longitudinal strain measured on apical three chambers; AP4 Strain: peak longitudinal strain measured on apical four chambers, BMI: body mass index; EF: ejection fraction; GDM: gestational diabetes mellitus; GLS: global peak longitudinal strain; GWG: gestational weight gain; IVS: ventricle interventricular septal; IVSs: ventricle interventricular septal in systole; IVSd: ventricle interventricular septal in diastole; LVH: left ventricle hypertrophy; LVMI: LV mass index; LVPWd: LV posterior wall in diastole; LVPWs: LV posterior wall in systole: LVDd: LV diameter in diastole; LVDs: LV diameter in systole; RWT: relative wall thickness.
