# Supplementary material for: Association of Maternal Gestational Weight Gain With Left Ventricle Geometry and Function in Offspring at 4 Years of Age: A Prospective Birth Cohort Study
Source: Front Pediatr. 2021 Aug 27;9:722385. doi: 10.3389/fped.2021.722385 (PMC8429845; doi:10.3389/fped.2021.722385)
Supplement: Supplementary file 3 [file Table_3.docx]

**Supplementary Table 3. Association between maternal GWG and offspring LV geometry and function in different gestational trimesters**

|  | Total GWG | First trimester GWG | | Second and third trimesters GWG |
| --- | --- | --- | --- | --- |
| LV Structure |  |  |  | |
| LVMI | 0.079(-0.024,0.184) | -0.059(-0.227,0.109) | **0.147(0.027,0.266)** | |
| LVPWs | **0.014(0.002,0.027)** | 0.017(-0.004,0.037) | 0.011(-0.004,0.026) | |
| LVPWd | **0.009(0.001,0.017)** | 0.011(-0.002,0.024) | 0.006(-0.003,0.016) | |
| LVDs | **0.027(0.000,0.054)** | 0.018(-0.025,0.062) | 0.030(-0.002,0.061) | |
| LVDd | **0.035(0.001,0.070)** | 0.002(-0.054,0.058) | **0.051(0.011,0.091)** | |
| IVSs | **0.022(0.009,0.035)** | -0.006(-0.027,0.015) | **0.033(0.017,0.047)** | |
| IVSd | **0.010(0.003,0.017)** | -0.001(-0.013,0.001) | **0.014(0.006,0.022)** | |
| RWT | **0.000(0.000,0.001)** | 0.000(-0.001,0.001) | 0.000(-0.001,0.001) | |
| LV Function |  |  |  | |
| E/a | -0.001(-0.005,0.004) | -0.001(-0.007,0.006) | 0.000(-0.004,0.005) | |
| Tei Index | 0.001(-0.000,0.001) | -0.000(-0.001,0.001) | 0.001(-0.000,0.002) | |
| EF | -0.013(-0.099,0.074) | -0.006(-0.129,0.140) | -0.020(-0.117,0.076) | |
| AP2 strain | -0.016(-0.068,0.036) | -0.003(-0.084,0.078) | -0.018(-0.076,0.041) | |
| AP3 strain | -0.042(-0.102,0.019) | -0.013(-0.107,0.082) | -0.045(-0.113,0.023) | |
| AP4 strain | 0.005(-0.046,0.055) | 0.070(-0.009,0.148) | -0.029(-0.086,0.027) | |
| GLS | -0.019(-0.061,0.024) | 0.017(-0.049,0.084) | -0.031(-0.079,0.016) | |

The bold values mean the mean difference is significant (P>0.05).

Data are presented as mean difference (95%CI).

AP2 Strain: peak longitudinal strain measured on apical two chambers; AP3 Strain: peak longitudinal strain measured on apical three chambers; AP4 Strain: peak longitudinal strain measured on apical four chambers, BMI: body mass index; EF: ejection fraction; GDM: gestational diabetes mellitus; GLS: global peak longitudinal strain; GWG: gestational weight gain; IVS: ventricle interventricular septal; IVSs: ventricle interventricular septal in systole; IVSd: ventricle interventricular septal in diastole; LVH: left ventricle hypertrophy; LVMI: LV mass index; LVPWd: LV posterior wall in diastole; LVPWs: LV posterior wall in systole: LVDd: LV diameter in diastole; LVDs: LV diameter in systole; RWT: relative wall thickness.
